# Activation of the Nrf2/ARE signaling pathway protects against palmitic acid-induced renal tubular epithelial cell injury by ameliorating mitochondrial reactive oxygen species-mediated mitochondrial dysfunction

**DOI:** 10.3389/fmed.2022.939149

**Published:** 2022-09-13

**Authors:** Xu-shun Jiang, Meng-yao Cai, Xun-jia Li, Qing Zhong, Man-li Li, Yun-feng Xia, Qing Shen, Xiao-gang Du, Hua Gan

**Affiliations:** ^1^Department of Nephrology, The First Affiliated Hospital of Chongqing Medical University, Chongqing, China; ^2^The Chongqing Key Laboratory of Translational Medicine in Major Metabolic Diseases, The First Affiliated Hospital of Chongqing Medical University, Chongqing, China

**Keywords:** Nrf2, palmitic acid, mitochondrial ROS, mitochondrial dysfunction, renal tubular epithelial cell

## Abstract

Chronic kidney disease (CKD) is often accompanied by dyslipidemia, and abnormal lipid metabolism in proximal tubule cells is considered closely related to the dysfunction of proximal tubule cells and eventually leads to accelerated kidney damage. Nuclear factor E2-related factor 2 (Nrf2), known as a redox-sensitive transcription factor, is responsible for regulating cellular redox homeostasis. However, the exact role of Nrf2 in dyslipidemia-induced dysfunction of proximal tubule cells is still not fully elucidated. In the present study, we showed that palmitic acid (PA) induced mitochondrial damage, excessive mitochondrial reactive oxygen species (ROS) (mtROS) generation, and cell injury in HK-2 cells. We further found that mtROS generation was involved in PA-induced mitochondrial dysfunction, cytoskeletal damage, and cell apoptosis in HK-2 cells. In addition, we demonstrated that the Nrf2/ARE signaling pathway was activated in PA-induced HK-2 cells and that silencing Nrf2 dramatically aggravated PA-induced mtROS production, mitochondrial damage, cytoskeletal damage and cell apoptosis in HK-2 cells. However, the mitochondrial antioxidant MitoTEMPOL effectively eliminated these negative effects of Nrf2 silencing in HK-2 cells under PA stimulation. Moreover, activation of the Nrf2/ARE signaling pathway with tBHQ attenuated renal injury, significantly reduced mtROS generation, and improved mitochondrial function in rats with HFD-induced obesity. Taken together, these results suggest that the Nrf2/ARE-mediated antioxidant response plays a protective role in hyperlipidemia-induced renal injury by ameliorating mtROS-mediated mitochondrial dysfunction and that enhancing Nrf2 antioxidant signaling provides a potential therapeutic strategy for kidney injury in CKD with hyperlipidemia.

## Introduction

Dyslipidemia is the most prevalent condition in patients with all stages of chronic kidney disease (CKD), including diabetic nephropathy (DN) (1, 2). The plasma levels of long-chain free fatty acids (FFAs) are chronically elevated in DN (3), and recently, elevated levels of urinary FFAs have been detected in patients with DN (4). The accumulation of intracellular fatty acids and their metabolites in non-adipose tissues can cause lipotoxicity, resulting in significant cellular dysfunction and injury (5). However, the mechanisms underlying the pathogenesis of lipid disorders accelerate the progression of CKD, particularly the role of hyperlipidemia in renal tubular epithelial cell injury, are still not fully understood.

Nuclear factor E2-related factor 2 (Nrf2), known as a redox-sensitive transcription factor, is responsible for regulating the cellular antioxidant response by modulating many detoxifying enzymes, phase II antioxidants, and stress-responsive proteins (6). Under rest conditions, Kelch ECH associated protein 1 (Keap1), as a repressor protein, interacts with Nrf2 to form a complex in the cytoplasm, which mediates Nrf2 degradation in the ubiquitin-proteasome system. Under cellular stress conditions, Nrf2 is released from Keap1, escapes proteasomal degradation, and translocates into the nucleus. In the nucleus, Nrf2 binds to the antioxidant response element (ARE) to trigger the transcription of downstream target genes such as haem oxygenase-1 (HO-1) and NADPH quinone oxidoreductase-1 (NQO-1) (7). This binding thus allows Nrf2 to efficiently provoke a cytoprotective response against cellular damage from a variety of reactive toxicants. Previous studies have demonstrated that Nrf2 signaling efficiently counteracts the production of ROS and is critical for maintaining the redox balance in the cell (8).

Accumulating evidence has demonstrated that the Nrf2/ARE pathway plays a major role in the pathogenesis of many diseases, including inflammatory diseases (9), atherosclerosis (10), cardiovascular disease (11), and neurodegenerative diseases (12), as well as kidney diseases, such as acute kidney injury (AKI) (13, 14), CKD (15) and DN (16). Shokeir et al. showed that the Nrf2 signaling pathway was activated and initiated a protective response against renal ischemia/reperfusion injury (13). In addition, Nrf2 was demonstrated to be crucial in ameliorating streptozotocin-induced renal damage by reducing hyperglycemia-induced oxidative stress (17, 18), indicating a beneficial effect of Nrf2 on DN. However, the function and regulation of Nrf2/ARE antioxidant signaling pathways in the development of CKD patients with dyslipidemia remain largely unknown.

This study aimed to investigate the role of the Nrf2/ARE antioxidant signaling pathways and clarify the possible mechanisms underlying the regulation of mitochondrial reactive oxygen species (mtROS) generation and mitochondrial function in hyperlipidemia-induced renal tubular epithelial cell injury.

## Materials and methods

### Cell culture and transfection

HK-2 cells (human proximal tubular epithelial cells) were a kind gift from Professor Ruan (The Centre for Nephrology, Royal Free and University College Medical School, London, United Kingdom). The cells were grown in DMEM/F-12 medium supplemented with 10% fetal bovine serum (FBS, Gibco) in an incubator with 5% CO2 at 37°C. For the knockdown of Nrf2, HK-2 cells were transiently transfected with Nrf2 siRNA using Lipofectamine 2000 (Invitrogen, Carlsbad, CA, United States) according to the manufacturer’s instructions. Nrf2 siRNA (#1 F: 5′-GGGAGGAGCUAUUAUCCAUTT-3′, R: 5′-AUGGAUAAUAGCUCCUCCCTT-3′; #2 F: 5′-GCCCAUU GAUGUUUCUGAUTT-3′, R: 5′-AUCAGAAACGAAAUCAA UGGGCTT-3′; #3 F: 5′-GCCUGUAAGUCCUGGUCAUTT-3′, R: 5′-AUGACCAGGACUUACAGGCTT-3′) and scrambled siRNA (F: 5′-UUCUCCGAACGUGUCACGUTT-3′, R: 5′-ACGUGACACGUUCGGAGAATT-3′) were constructed by GenePharma Company (Shanghai, China).

### Animals

Male Sprague-Dawley (SD) rats (4 weeks old) were provided by the Animal Center of Chongqing Medical University and kept under a 12-h light/12-h dark cycle. After 1 week of acclimatization, 24 rats were randomly divided into the following three groups with eight animals in each group: the normal control group (low-fat diet [LFD] group), which was given a normal chow diet (#D12450B; 3.85 kcal/g, 10% from fat; Research Diets, New Brunswick, NJ, United States); the high-fat diet (HFD) group, which was given a western high-fat diet (#D12451; 4.73 kcal/g, 45% from fat; Research Diets); and the HFD group, which received an intraperitoneal injection of tert-butylhydroquinone (tBHQ) (Sigma–Aldrich, 50 mg/kg, every other day for 12 weeks). The rats were weighed and then sacrificed at 20 weeks of age. Blood was collected into chilled tubes and subsequently centrifuged (4°C, 3000 rpm, 15 min) to obtain plasma, which was then stored at –80°C for further analysis. Kidney tissues were placed in liquid nitrogen for Western blot analysis and immunofluorescence staining or fixed in formalin and embedded in paraffin for H&E and PAS staining. All studies were approved by the Ethics Committee of Chongqing Medical University.

### Assessment of lipid accumulation

To evaluate lipid accumulation, frozen kidney sections and HK-2 cells were fixed with 4% formaldehyde for 15 min. After fixation, kidney tissues and HK-2 cells were washed three times with phosphate buffered saline (PBS) and then stained with oil red O solution for 30 min at room temperature. After washing three times with PBS to remove the excess oil red O dye, the kidney tissues and HK-2 cells were pretreated with 60% isopropanol and then stained with hematoxylin for 5 min. Additionally, to confirm lipid accumulation in kidney tissues and HK-2 cells, BODIPY lipid probe staining (10 μg/ml, BODIPY500/510 C1, C12, Invitrogen, Carlsbad, CA, United States) was performed. Briefly, kidney tissues and HK-2 cells were stained with BODIPY lipid probe (10 μg/ml) at 37°C for 1 h in the dark and then counterstained with 4,6-diamidino-2-phenylindole (DAPI) for 3 min. Images were observed under a fluorescence microscope.

### Determination of mitochondrial reactive oxygen species production

Intercellular mitochondrial ROS production in HK-2 cells was detected using the fluorescent reagent MitoSOX Red (M36008, Invitrogen, Carlsbad, CA, United States). Briefly, after treatment, HK-2 cells were incubated with MitoSOX Red (5 μM) and MitoTracker green (50 nM, C1048, Beyotime, China) at 37°C for 20 min in the dark, or the treated cells were incubated with DCFH-DA (10 μM) and MitoTracker Red (50 nM) at 37°C for 30 min in the dark. For kidney tissue, 4-μm-thick frozen sections were stained with 5 μM MitoSOX Red at 37°C for 20 min in the dark, followed by washing three times with PBS. The images were analyzed under a fluorescence microscope.

### Detection of mitochondrial transmembrane potential (ΔΨm)

HK-2 cells were seeded in 12-well plates. After treatment, the cells were washed three times with PBS, followed by incubation with JC-1 fluorescence dye (Beyotime, China) for 20 min at 37°C in the dark. For kidney tissue, 4-μm-thick frozen sections were also incubated with JC-1 fluorescence dye for 20 min at 37°C in the dark. After this short incubation, HK-2 cells and kidney tissue were washed three times with JC-1 staining buffer, and the fluorescence intensity of the cells or frozen kidney sections was observed under a fluorescence microscope.

### Measurement of oxidative stress markers

Kidney tissues were lysed and centrifuged, and the supernatants were collected for the measurement of superoxide dismutase (SOD), glutathione (GSH), and malondialdehyde (MDA) levels using the corresponding kits, according to the manufacturers’ protocol (Beyotime Institute of Biotechnology, China).

### Staining for actin cytoskeleton

HK-2 cells were seeded on coverslips in 12-well plates. After the indicated treatments, HK-2 cells were washed twice with PBS and fixed with 4% paraformaldehyde at room temperature for 20 min. The cells were then permeabilized with 0.1% Triton X-100 for 3 min and blocked with 5% bovine serum albumin (BSA) for 1 h. Subsequently, HK-2 cells were stained with Alexa594-phalloidin (Invitrogen, Carlsbad, CA, United States) for 30 min in the dark at room temperature. After washing, the cells were counterstained with DAPI and visualized with a fluorescence microscope.

### Western blot analysis

For immunoblot analysis, total protein was extracted by RIPA lysis buffer (Beyotime, China), and centrifuged at 12,000 *g* for 15 min at 4°C. Then, equal amounts of protein samples (30 μg per lane) from kidney or HK-2 cells were resolved on a 10–12% SDS-PAGE gel and then transferred onto PVDF membranes (Millipore) after electrophoresis. After blocking with 5% non-fat milk for 3 h at room temperature, the PVDF membranes were washed with TBST and then incubated overnight at 4°C with the following antibodies: rabbit anti-Nrf2 (1:1000, Abcam); rabbit anti-HO-1 (1:5000, Abcam); rabbit anti-NQO-1 (1:5000, Abcam); rabbit anti-cleaved-caspase3 (1:1000, CST); rabbit anti-cytochrome c (1:1000, CST); rabbit anti-Bax (1:1000, CST); rabbit anti-Bcl-2 (1:1000, CST); rabbit anti-PARP (1:1000, CST); rabbit anti-caspase 9 (1:1000, CST); and mouse anti-β-actin (1:5000, Sungene Biotech, Tianjin, China). Subsequently, the membranes were washed three times with TBST for 30 min and incubated with the appropriate secondary horseradish peroxidase (HRP)-conjugated antibodies for 1 h at room temperature. Immunoreactive bands were visualized by an ECL chemiluminescence system (GE Healthcare, Piscataway, NJ, United States), and densitometric analysis of the bands was performed using Quantity One software.

### Analysis of cell death and apoptosis

Cytotoxicity was estimated by a Cell Counting Kit-8 (CCK-8) colorimetric assay (Sigma-Aldrich, St. Louis, MO, United States). HK-2 cells were seeded onto a 96-well plate and treated with various concentrations of BSA or PA (Sigma, United States, P9767) (0, 100, 150, 200, 250, 300, 350, 400, 450 μM) for 24 h. Then, the treated cells were incubated with a CCK-8 kit (100 μL/well) for 2 h at 37°C in a 5% CO2 incubator. After the plate was agitated, the absorbances of each well at 450 nm were measured using a multiwell fluorescence plate reader (Thermo Scientific Varioskan Flash).

To evaluate the extent of cell apoptosis, an Annexin V-FITC/PI apoptosis assay kit (Sungene Biotech, Tianjin, China) was used to assess cell apoptosis. Briefly, after the indicated treatments, the cells were stained with 10 μg/ml Annexin V/FITC for 30 min at 37°C and with 5 μg/ml propidium iodide (PI) for 5 min at room temperature, followed by flow cytometry analysis using a BD FACSVantage SE cytometer.

### Immunofluorescent staining

HK-2 cells were seeded in 12-well plates, and following the indicated treatments, the cells were stained with 50 nM MitoTracker Red in medium for 30 min. After washing, the cells were fixed with 4% paraformaldehyde for 20 min followed by permeabilization with 0.1% Triton X-100 for 3 min. The cells were then blocked with 5% BSA for 1 h and incubated with primary antibodies against Nrf2 (1:200, #31163, Abcam), HO-1 (1:200, #68477, Abcam), or NQO-1 (1:200, #80588, Abcam) at 4°C overnight. After washing three times with PBS, the cells were incubated with the appropriate fluorophore-conjugated secondary antibody for 1.5 h in the dark. Subsequently, the cells were counterstained with DAPI and visualized with a fluorescence microscope.

For kidney tissue, 4-μm-thick frozen sections were rewarmed at room temperature and then fixed with 4% paraformaldehyde for 20 min followed by permeabilization with 0.1% Triton X-100 for 3 min. After washing, the frozen sections were blocked with 5% BSA at room temperature for 1 h and then incubated with primary antibodies against Nrf2 (1:200, #31163, Abcam), HO-1 (1:200, #68477, Abcam), or NQO-1 (1:200, #80588, Abcam) at 4°C overnight. After washing three times with PBS, the slides were incubated with the appropriate fluorophore-conjugated secondary antibody for 1.5 h in the dark and then stained with DAPI for 3 min. Finally, the slides were detected by fluorescence microscopy. The relative intensity of immunofluorescence was quantified using Image-Pro Plus software.

### Transmission electronic microscopy

Fresh kidney tissue was harvested in 1-mm^3^ pieces and then fixed with 3% glutaraldehyde at 4°C overnight. After postfixation in 1% osmium tetroxide at room temperature for 2 h, the kidney tissue was dehydrated in a graded series of ethanol and embedded in epoxy resin. Ultrathin sections were stained with 7% uranyl acetate and 1% lead citrate and examined under a JEM-1200 EXII transmission electron microscope (JEOL, Tokyo, Japan).

### Statistical analysis

Quantitative data are presented as the mean ± SEM. Statistical differences between the 2 groups were analyzed using Student’s *t*-test, and multiple comparisons were analyzed with one-way analysis of variance (ANOVA) followed by Tukey’s *post hoc* test. *P* < 0.05 was considered significant.

## Results

### Palmitic acid induced lipid accumulation and cell injury in HK-2 cells

**FIGURE 1:**
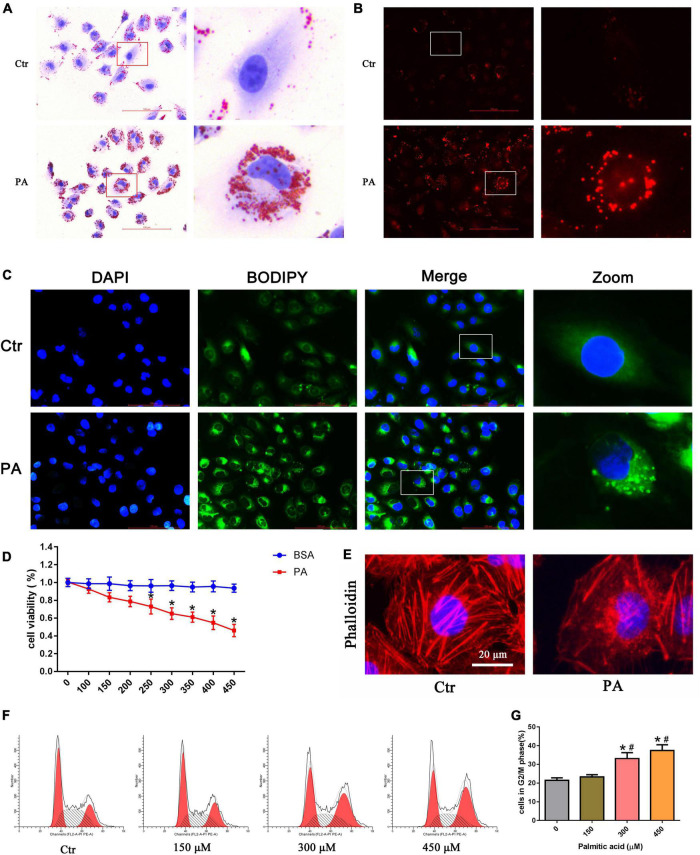
Palmitic acid (PA) induced lipid accumulation and cell injury in HK-2 cells. **(A–C)** Intracellular lipid accumulation in HK-2 cells after treatment with 300 μmol/L PA for 24 h was detected by oil red O staining **(A)**, Nile red staining **(B)** and BODIPY lipid probe (400×) **(C)**. **(D)** HK-2 cells were treated with different concentrations of PA or BSA for 24 h, and viable cells were quantified using the CCK-8 assay. (*n* = 3, *P < 0.05 vs. the BSA treatment group). **(E)** HK-2 cells were treated with 300 μmol/L PA for 24 h and then visualized by fluorescence-labeled phalloidin staining. **(F)** Representative images of flow cytometry analysis of the cell cycle after HK-2 cells were treated with different concentrations of PA for 24 h. **(G)** Percentage of cells in the G2/M phase in **(F)**. (*n* = 3, *P < 0.05 vs. 0 μmol/L, ^#^*P* < 0.05 vs. 150 μmol/L).

Using oil red O, Nile Red, and BODIPY lipid probe staining, we confirmed that palmitic acid (PA) induced obvious intracellular lipid accumulation in HK-2 cells (Figures 1A–C). Cytotoxic accumulation of lipids in non-adipose tissues can lead to cellular lipotoxicity. To examine whether PA treatment could affect cell viability, we administered various concentrations of PA (0–450 μM) to HK-2 cells for 24 h, and then the CCK-8 assay was performed. As indicated in Figure 1D, PA decreased HK-2 cell viability in a dose-dependent manner.

In addition, to observe the effect of PA on cytoskeletal changes, HK-2 cells were stained with fluorescence-labeled phalloidin. As shown in Figure 1E, control cells exhibited well-organized stress fibers throughout the cytoplasm, and the cytoskeleton was clearly visible and remained intact. PA induced disorganization and rearrangement and decreased actin stress fibers in HK-2 cells. Furthermore, we used flow cytometry to investigate the effects of PA on the cell cycle. As shown in Figures 1F,G, PA treatment for 24 h dose-dependently induced the accumulation of HK-2 cells in the G2/M phase.

### Palmitic acid induced HK-2 cell mitochondrial damage and mitochondrial reactive oxygen species production and activated the intrinsic apoptotic pathway

**FIGURE 2:**
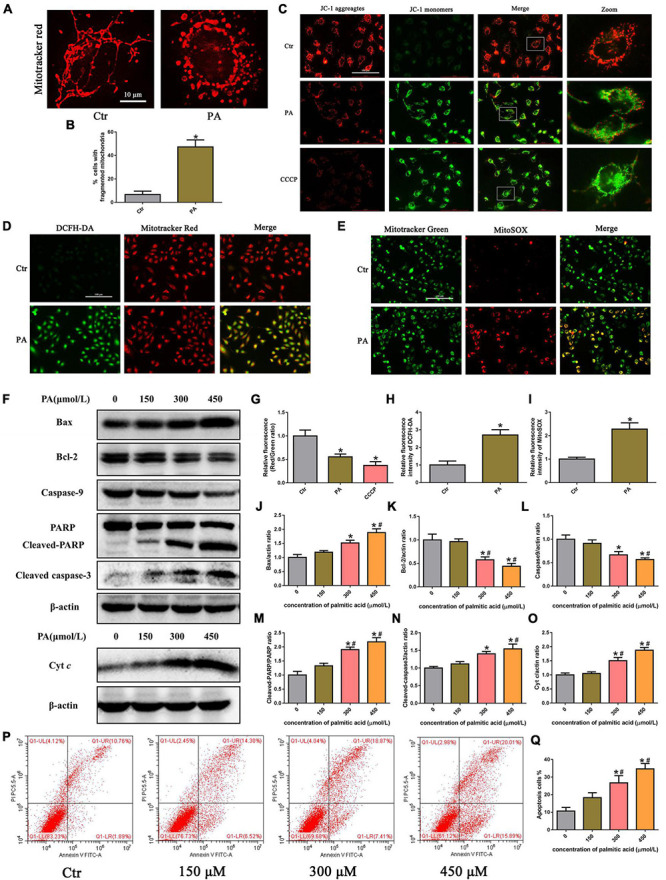
Palmitic acid (PA) induced HK-2 cell mitochondrial damage and mitochondrial ROS production and activated the intrinsic apoptotic pathway. **(A)** Treatment with 300 μmol/L PA for 24 h induced more fragmented mitochondria in HK-2 cells, as shown by staining with MitoTracker Red. **(B)** Quantitative image analysis of the percentage of cells with fragmented mitochondria. **(C)** HK-2 cells were treated with 300 μmol/L PA for 24 h or 10 μM CCCP for 1 h, and the ΔΨm of cells was detected by JC-1 fluorescence dye. **(D)** Representative immunofluorescence microscopic images of intracellular ROS in HK-2 cells (200×). HK-2 cells were treated with 300 μmol/L PA for 24 h, and the cells were then stained with 10 μM DCFH-DA (green) and 50 nM MitoTracker (red). **(E)** HK-2 cells were treated with 300 μmol/L palmitic acid for 24 h, and then the cells were stained with 5 μM MitoSOX (red) and 50 nM MitoTracker (green). Colocalization of MitoTracker and MitoSOX supports that superoxide is likely generated by mitochondria (400×). **(F)** Representative Western blot analyses of Bax, Bcl-2, Caspase9, PARP, cleaved-caspase3, and Cyt *c* expression in HK-2 cells treated with different concentrations of PA for 24 h. **(G–I)** Relative fluorescence intensities of JC-1, DCFH-DA, or MitoSOX from three randomly selected microscopic fields per group were measured and analyzed. **(J–O)** Densitometric analysis of Bax, Bcl-2, Caspase9, PARP, cleaved-caspase3, and Cyt *c* expression in **(F)**. **(P)** Representative images of flow cytometry analysis of cell apoptosis after HK-2 cells were treated with different concentrations of PA for 24 h. **(Q)** Percentage of apoptotic cells in **(P)**. (*n* = 3, *P < 0.05 vs. the control group, ^#^*P* < 0.05 vs. 150 μmol/L, ^Δ^*P* < 0.05 vs. 300 μmol/L).

To examine mitochondrial morphology under PA stimulation, HK-2 cells were stained with MitoTracker Red. As shown in Figures 2A,B, mitochondria in control cells exhibited an intact mitochondrial network, whereas they transformed into short rods after PA (300 μM) treatment for 24 h. PA stimulated mitochondrial membrane potential (ΔΨm) collapse in HK-2 cells as assessed by JC-1 staining, as shown in Figure 2C. Fluorescence analysis showed that, similar to CCCP, a powerful mitochondrial uncoupling agent that has been implicated in mitochondrial depolarization, PA induced more diffuse J-monomer fluorescence staining and less J-aggregate staining than that observed in the control group. A fluorescence intensity analysis showed that the red/green fluorescence intensity ratio was significantly decreased in the PA group, suggesting that PA caused a decrease in ΔΨm and mitochondrial damage in HK-2 cells (Figure 2G).

Mitochondria are the major source of ROS in cells, especially damaged mitochondria, which can release significant amounts of ROS into the cytosol and cause intracellular oxidative stress (19). To determine the effect of PA on ROS generation in HK-2 cells, we used the ROS-sensitive fluorescent probe DCFH-DA to monitor cellular oxidative stress. As shown in Figures 2D,H, PA exposure dramatically increased ROS production in HK-2 cells, and the overlapping staining for ROS and mitochondria suggests that mtROS production was induced by PA. Moreover, we confirmed that PA enhanced mtROS generation and colocation to mitochondria in HK-2 cells by immunofluorescence assays, using MitoSOX Red to quantify mitochondrial ROS production and MitoTracker green to label total mitochondria (Figures 2E,I).

The Bcl-2 family of proteins plays an important role in regulating mitochondrial function, and mitochondrial damage facilitates cytochrome c release from mitochondria into the cytoplasm, which leads to activation of the caspase cascade and mitochondria-mediated apoptosis (20). As shown in Figures 2F,P,Q, an increase in apoptosis was observed in HK-2 cells subjected to PA, as determined by flow cytometry. Apoptosis was accompanied by the dose-dependent upregulation of Bax and cytochrome c, cleaved caspase-3, and cleaved poly (ADP-ribose) polymerase (PARP) and concomitant with the decreased expression of Bcl-2 and caspase-9 (Figures 2I–O). These results indicated that PA induced HK-2 cell mitochondrial damage and mitochondrial ROS production and activated the intrinsic apoptotic pathway.

### Role of mitochondrial reactive oxygen species in palmitic acid-induced mitochondrial dysfunction and cell injury in HK-2 cells

**FIGURE 3:**
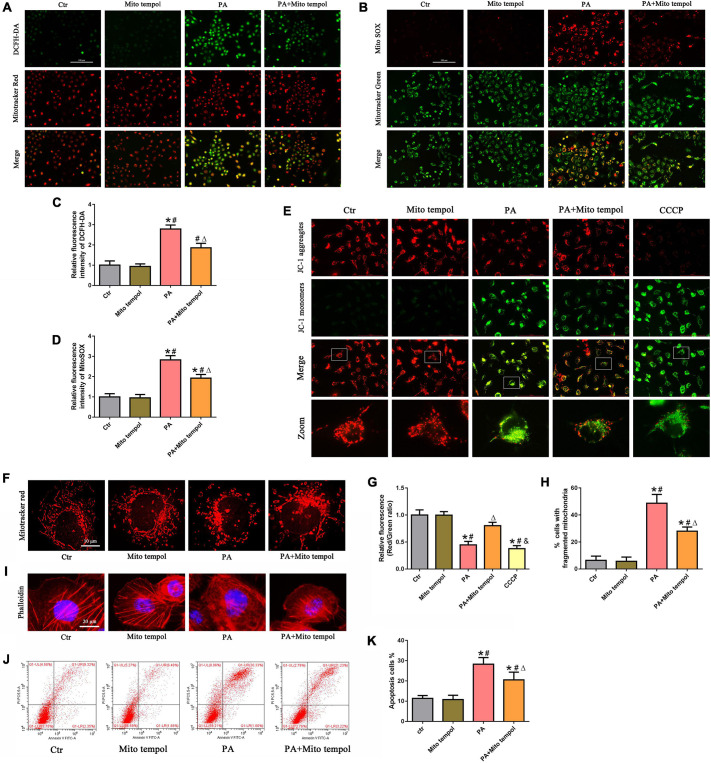
Role of mitochondrial ROS in PA-induced mitochondrial dysfunction and cell injury in HK-2 cells. **(A)** HK-2 cells were treated with 300 μmol/L PA for 24 h after pretreatment with or without MitoTEMPOL (100 nM) for 1 h and then stained with DCFH-DA (green) and MitoTracker (red). **(B)** HK-2 cells were treated with 300 μmol/L PA for 24 h after pretreatment with or without MitoTEMPOL for 1 h and then stained with MitoSOX (red) and MitoTracker (green). **(C,D)** Relative fluorescence intensities of DCFH-DA or MitoSOX from three randomly selected microscopic fields per group were measured and analyzed. **(E,F)** HK-2 cells were treated with 300 μmol/L PA for 24 h after pretreatment with or without MitoTEMPOL for 1 h, and the cells were then stained with JC-1 fluorescence dye or MitoTracker (red) (400×). **(G)** Relative fluorescence intensities of JC-1 from three randomly selected microscopic fields per group were measured and analyzed. **(H)** Quantitative image analysis of the percentage of cells with fragmented mitochondria. **(I)** HK-2 cells were treated with 300 μmol/L PA for 24 h after pretreatment with or without MitoTEMPOL for 1 h and then visualized by fluorescence-labeled phalloidin staining. **(J)** HK-2 cells were treated with 300 μmol/L PA for 24 h after pretreatment with or without MitoTEMPOL for 1 h, and cell apoptosis was analyzed by flow cytometry. **(K)** Percentage of apoptotic cells in **(J)**. (*n* = 3, *P < 0.05 vs. the control group, ^#^*P* < 0.05 vs. the MitoTEMPOL group, ^Δ^*P* < 0.05 vs. the PA group, ^&^*P* < 0.05 vs. the PA + MitoTEMPOL group).

To investigate the role of mitochondrial ROS generation in PA-induced mitochondrial dysfunction, HK-2 cells were preincubated with MitoTEMPOL, a derivative of the antioxidant tempol that is specifically targeted to the mitochondria. As shown in Figures 3A–D, we found that MitoTEMPOL significantly attenuated the PA-induced increase in mitochondrial ROS production and partially reversed the loss of PA-induced ΔΨm (Figures 3E,G). Meanwhile, MitoTEMPOL treatment also significantly attenuated PA-induced mitochondrial fragmentation (Figures 3F,H). Moreover, MitoTEMPOL treatment significantly ameliorated PA-induced cytoskeletal changes (Figure 3I) and cell apoptosis (Figures 3J,K). These results demonstrated a significant role for mtROS-mediated mitochondrial dysfunction and cell injury in PA-induced HK-2 cells.

### High lipid induced activation of the Nrf2/ARE signaling pathway in HK-2 cells

**FIGURE 4:**
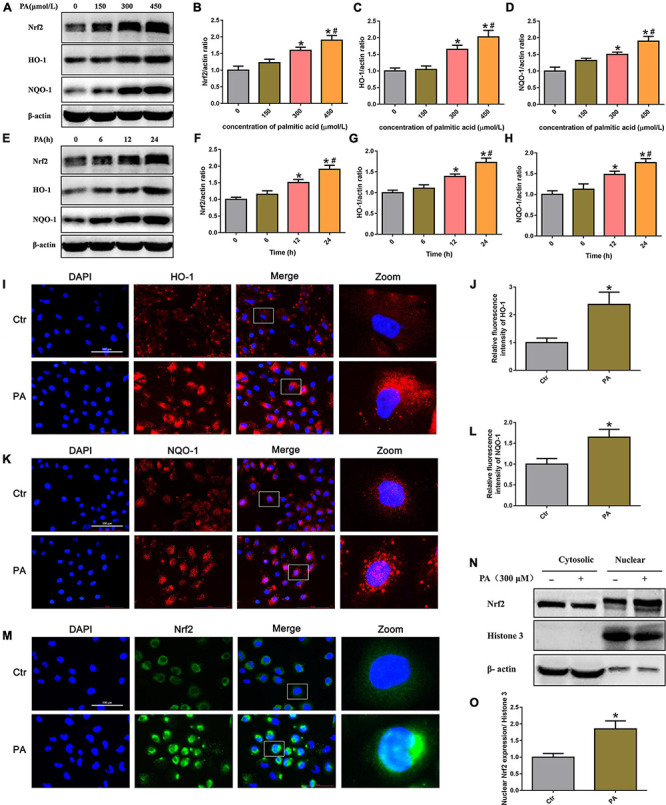
High lipid-induced activation of the Nrf2/ARE signaling pathway in HK-2 cells. **(A)** Representative Western blots of the expression of Nrf2, HO-1, and NQQ-1 after HK-2 cells were treated with PA for 24 h at the indicated concentrations. **(B–D)** Densitometric analysis of Nrf2, HO-1, and NQQ-1 expression in **(A)**. (*n* = 3, *P < 0.05 vs. 0 μmol/L, ^#^*P* < 0.05 vs. 150 μmol/L, ^Δ^*P* < 0.05 vs. 300 μmol/L). **(E)** Representative Western blots of the expression of Nrf2, HO-1, and NQQ-1 after HK-2 cells were treated with 300 μmol/L PA at the indicated time points. **(F–H)** Densitometric analysis of Nrf2, HO-1, and NQQ-1 expression in panel E. (*n* = 3, *P < 0.05 vs. 0 h, ^#^*P* < 0.05 vs. 6 h, ^Δ^*P* < 0.05 vs. 12 h). **(I)** HK-2 cells were treated with 300 μmol/L PA for 24 h and then stained with an antibody against HO-1 (400×). **(J)** Relative fluorescence intensities of HO-1 from three randomly selected microscopic fields per group were measured and analyzed. (*n* = 3, *P < 0.05 vs. control group). **(K)** HK-2 cells were treated with 300 μmol/L PA for 24 h and then stained with an antibody against NQQ-1. **(L)** Relative fluorescence intensities of NQO-1 from three randomly selected microscopic fields per group were measured and analyzed. *P < 0.05 vs. control group. **(M)** HK-2 cells were treated with 300 μmol/L PA for 24 h and then stained with an antibody against Nrf2 (400×). **(N)** Representative Western blot analyses of Nrf2 nuclear translocation in HK-2 cells after nuclear fractionation. **(O)** Densitometric analysis of nuclear Nrf2 expression in **(N)**. (*n* = 3, *P < 0.05 vs. the control group).

To determine whether PA exposure activates the Nrf2/ARE signaling pathway in HK-2 cells, we initially treated HK-2 cells with different concentrations of PA and found that Nrf2, HO-1, and NQO-1 protein expression was markedly increased in a dose-dependent manner (Figures 4A–D). In addition, we treated HK-2 cells with 300 μmol/L PA and found that the expression of Nrf2, HO-1, and NQQ-1 in HK-2 cells increased in a time-dependent manner, as demonstrated by Western blot analysis (Figures 4E–H). By immunofluorescence, we showed a clear increase in the fluorescence intensity of HO-1 and NQQ-1 in PA-induced HK-2 cells compared with control cells (Figures 4I–L). Next, we detected the distribution of Nrf2 in HK-2 cells and found that Nrf2 was located primarily in the cytoplasm in the control cells, while it was predominantly located in the nucleus and accompanied by enhanced fluorescence intensity in the treated cells (Figure 4M). Nrf2 nuclear translocation was further confirmed by Western blotting of HK-2 cells after nuclear fractionation (Figure 4N). As shown in Figure 4O, there was approximately twice as much Nrf2 protein in the nuclear compartment and correspondingly less Nrf2 in the cytosolic compartment of PA-induced HK-2 cells compared with the control cells. These findings confirm that PA activates the Nrf2/ARE pathway and upregulates its target enzymes.

### Silencing of Nrf2 accelerated palmitic acid-induced mitochondrial reactive oxygen species generation in HK-2 cells

**FIGURE 5:**
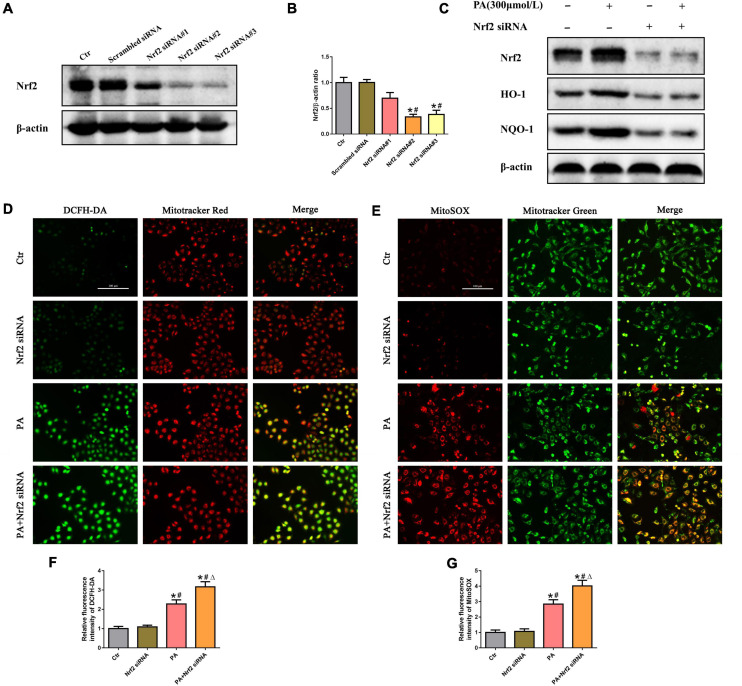
Silencing of Nrf2 accelerated PA-induced mtROS generation in HK-2 cells. **(A)** Western blot analysis of Nrf2 in HK-2 cells after transfection with or without scrambled siRNA or Nrf2 siRNA. **(B)** Densitometric analysis of Nrf2 expression in **(A)**. (*n* = 3, *P < 0.05 vs. the control group, ^#^*P* < 0.05 vs. the scrambled siRNA group, ^Δ^*P* < 0.05 vs. the Nrf2 siRNA#1 group). **(C)** Western blot analysis of Nrf2, HO-1, and NQO-1 in PA-induced HK-2 cells after transfection with or without Nrf2 siRNA. **(D,E)** HK-2 cells were treated with 300 μmol/L PA for 24 h after transfection with or without Nrf2 siRNA, and then the cells were stained with DCFH-DA (green) and MitoTracker (red) or incubated with MitoSOX (red) and MitoTracker (green). **(F,G)** Relative fluorescence intensities of DCFH-DA or MitoSOX from three randomly selected microscopic fields per group were measured and analyzed. (*n* = 3, *P < 0.05 vs. the control group, ^#^*P* < 0.05 vs. the Nrf2 siRNA group, ^Δ^*P* < 0.05 vs. the PA group).

To investigate the effects of Nrf2 on PA-induced mtROS generation in HK-2 cells, siRNA targeting Nrf2 was used to knock down endogenous Nrf2 expression. As shown in Figures 5A,B, compared with the control, Nrf2 siRNA#2 and #3 significantly suppressed Nrf2 protein levels. We thus selected Nrf2 siRNA#2 to perform the following experiments and confirmed that knockdown of Nrf2 markedly decreased the protein levels of its downstream targets HO-1 and NQQ-1 in HK-2 cells by Western blot analysis (Figure 5C). Furthermore, we found that silencing Nrf2 further enhanced PA-induced mtROS production (Figures 5D–G).

### Silencing of Nrf2 accelerated palmitic acid-induced mitochondrial dysfunction and cell injury in HK-2 cells

**FIGURE 6:**
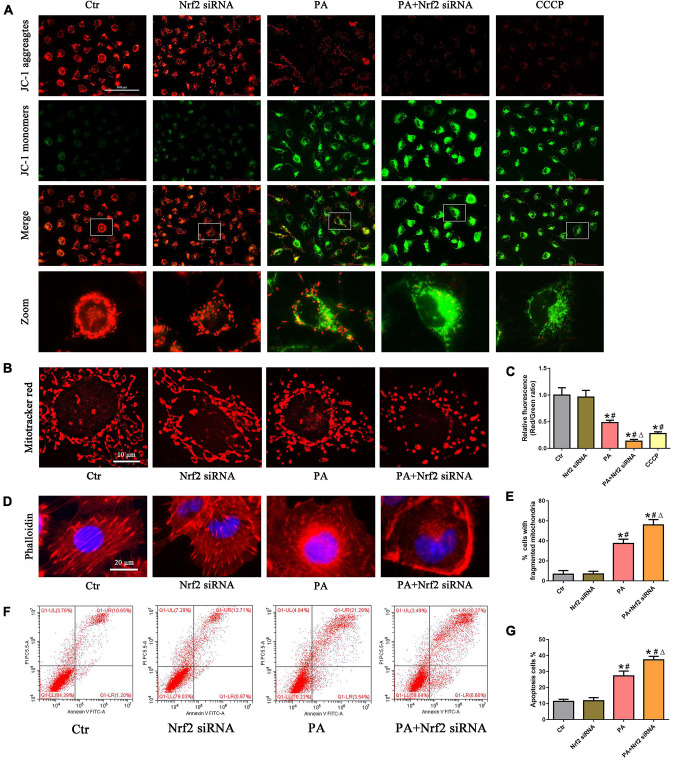
Silencing of Nrf2 accelerated PA-induced mitochondrial dysfunction and cell injury in HK-2 cells. **(A,B)** HK-2 cells were treated with 300 μmol/L PA for 24 h after transfection with or without Nrf2 siRNA, and the cells were stained with JC-1 fluorescence dye or MitoTracker (red). **(C)** Relative fluorescence intensities of JC-1 from three randomly selected microscopic fields per group were measured and analyzed. **(D)** HK-2 cells were treated with 300 μmol/L PA for 24 h after transfection with or without Nrf2 siRNA, and the cells were visualized by fluorescence-labeled phalloidin staining. **(E)** Quantitative image analysis of the percentage of cells with fragmented mitochondria. **(F)** HK-2 cells were treated with 300 μmol/L PA for 24 h after transfection with or without Nrf2 siRNA, and cell apoptosis was analyzed by flow cytometry. **(G)** Percentage of apoptotic cells in **(F)**. (*n* = 3, *P < 0.05 vs. the control group, ^#^*P* < 0.05 vs. the Nrf2 siRNA group, ^Δ^*P* < 0.05 vs. the PA group).

We then investigated the role of the Nrf2/ARE signaling pathway in PA-induced mitochondrial dysfunction and cell injury in HK-2 cells and found that silencing Nrf2 further accelerated the loss of PA-induced ΔΨm (Figures 6A,C) and mitochondrial fragmentation (Figures 6B,E) in HK-2 cells. In addition, silencing Nrf2 further accelerated PA-induced cytoskeletal damage (Figure 6D) and cell apoptosis (Figures 6F,G).

### MitoTEMPOL alleviated the negative effect of Nrf2 silencing on palmitic acid-induced mitochondrial dysfunction and cell injury in HK-2 cells

**FIGURE 7:**
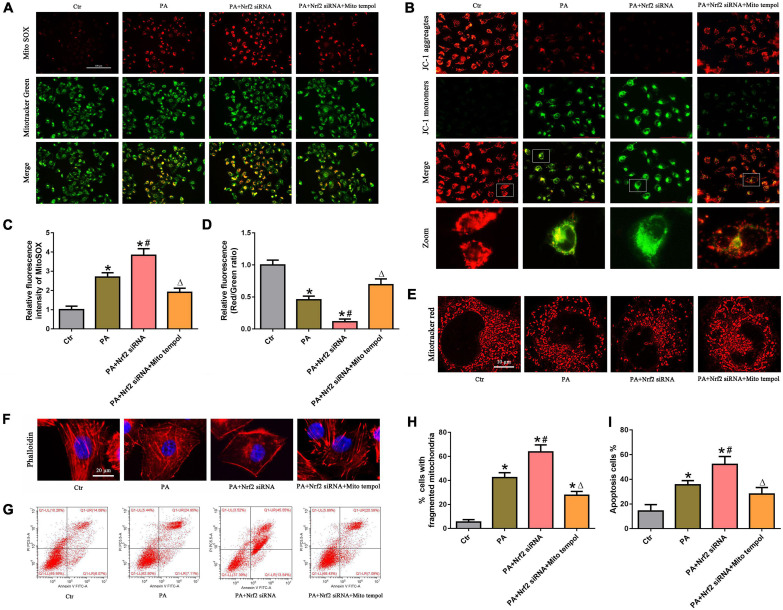
MitoTEMPOL alleviated the negative effect of Nrf2 silencing on PA-induced mitochondrial dysfunction and cell injury in HK-2 cells. **(A)** HK-2 cells were treated with 300 μmol/L PA for 24 h after transfection with Nrf2 siRNA and/or pretreatment with MitoTEMPOL, and the cells were stained with MitoSOX (red) and MitoTracker (green). **(B)** HK-2 cells were treated with 300 μmol/L PA for 24 h after transfection with Nrf2 siRNA and/or pretreatment with MitoTEMPOL, and the cells were then stained with JC-1 fluorescence dye. **(C,D)** Relative fluorescence intensities of MitoSOX or JC-1 from three randomly selected microscopic fields per group were measured and analyzed. (*n* = 3, *P < 0.05 vs. the control group, ^#^*P* < 0.05 vs. the Nrf2 siRNA group, ^Δ^*P* < 0.05 vs. the PA group). **(E,F)** HK-2 cells were treated with 300 μmol/L PA for 24 h after transfection with Nrf2 siRNA and/or pretreatment with MitoTEMPOL. Then, the cells were visualized by MitoTracker (red) or fluorescence-labeled phalloidin staining. **(G)** HK-2 cells were treated with 300 μmol/L PA for 24 h after transfection with Nrf2 siRNA and/or pretreatment with MitoTEMPOL, and cell apoptosis was analyzed by flow cytometry. **(H)** Quantitative image analysis of the percentage of cells with fragmented mitochondria in **(E)**. **(I)** Percentage of apoptotic cells in **(G)**. (*n* = 3, *P < 0.05 vs. the control group, ^#^*P* < 0.05 vs. the PA group, ^Δ^*P* < 0.05 vs. the PA + Nrf2 siRNA group).

To confirm the protective role of the Nrf2/ARE signaling pathway by ameliorating mtROS-mediated mitochondrial dysfunction, PA-stimulated HK-2 cells were pretreated with both NRF2 siRNA and MitoTEMPOL. As shown in Figures 7A,C, silencing Nrf2 further enhanced PA-induced mtROS generation. However, MitoTEMPOL significantly blocked NRF2 silencing-mediated mtROS production in PA-induced HK-2 cells. Moreover, MitoTEMPOL effectively eliminated the negative effect of Nrf2 silencing on ΔΨm (Figures 7B,D), mitochondrial fragmentation (Figures 7E,H), cytoskeletal damage (Figure 7F) and cell apoptosis (Figures 7G,I) in PA-induced HK-2 cells, indicating that the potential protective mechanism of the Nrf2/ARE signaling pathway on PA-induced HK-2 cells is involved in ameliorating mtROS-mediated mitochondrial dysfunction.

### Effect of the Nrf2 activator tBHQ on renal functional and morphologic characteristics in the kidneys of rats with high-fat diet-induced obesity

**FIGURE 8:**
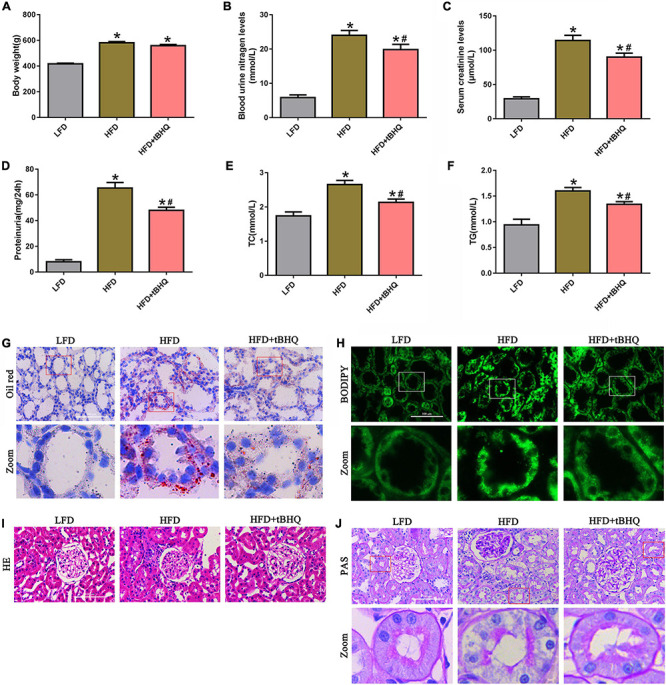
Effect of the Nrf2 activator tBHQ on renal functional and morphologic characteristics in the kidneys of rats with HFD-induced obesity. **(A)** Body weight changes in LFD, HFD, and HFD rats treated with tBHQ. **(B)** Blood urea nitrogen (BUN) levels. **(C)** Serum creatinine (SCr) levels. **(D)** Urine protein levels. **(E)** Serum total cholesterol (TC) levels. **(F)** Triglyceride (TG) levels. (*n* = 8, *P < 0.05 vs. the LFD group. ^#^*P* < 0.05 vs. the HFD group). **(G,H)** BODIPY and oil red O staining of kidney tissues in LFD, HFD rats, and HFD rats treated with tBHQ (400×). **(I,J)** Kidney sections stained with H&E PAS (400×).

We examined the role of the Nrf2/ARE pathway in the kidneys of rats with HFD-induced obesity. The HFD rats receiving tBHQ had a smaller reduction in body weight than the untreated HFD rats; however, there was no significant difference in body weight between the HFD rats treated with tBHQ and the untreated HFD rats (Figure 8A). Blood urea nitrogen (BUN), serum creatinine (SCr), urine protein, serum total cholesterol (TC), and triglyceride (TG) levels were increased in HFD rats compared with LFD rats; however, all of these increases were significantly attenuated following treatment with tBHQ (Figures 8B–F). Next, oil red O staining and BODIPY lipid probes showed significant lipid accumulation in proximal tubule cells in the kidney tissues of HFD rats (Figures 8G,H); however, tBHQ treatment significantly decreased the levels of lipid accumulation in proximal tubule cells in HFD rats. Moreover, H&E and PAS staining showed that glomerular mesangial matrix proliferation was enhanced in HFD rats compared with LFD rats; this enhancement was alleviated in HFD rats treated with tBHQ (Figures 8I,J). PAS staining also revealed notable tubular epithelial disruption in the HFD group. The HFD rats treated with tBHQ showed notable improvement in the tubular compartment with less cellular disruption (Figure 8J).

### Activation of the Nrf2/ARE signaling pathway ameliorated mitochondrial reactive oxygen species generation and mitochondrial injury in the kidneys of rats with high-fat diet-induced obesity

**FIGURE 9:**
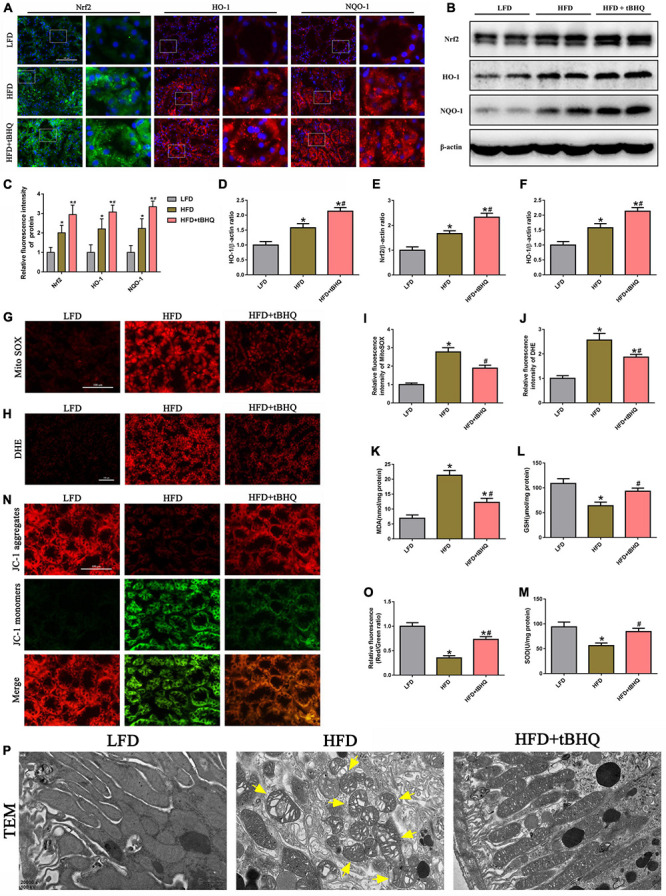
Activation of the Nrf2/ARE signaling pathway ameliorated mtROS generation and mitochondrial injury in the kidneys of rats with HFD-induced obesity. **(A)** Representative images of immunofluorescence staining and Western blot of Nrf2, HO-1, and NQQ-1 in kidney tissues. **(C)** Quantification of immunofluorescence staining of Nrf2, HO-1, and NQQ-1 in **(A)**. **(D–F)** Densitometric analysis of Nrf2, HO-1, and NQQ-1 expression in **(B)**. **(G,H)** Kidney sections stained with MitoSOX and DHE. **(I,J)** Relative fluorescence intensities of MitoSOX and DHE staining. **(K–M)** MDA content, GSH activity, and SOD activity in rat kidney tissue were determined. **(N)** Kidney sections stained with JC-1 (400×). **(O)** Relative fluorescence intensities of JC-1 staining. **(P)** Mitochondrial morphology of the tubular cells was observed by TEM (20,000×). Representative TEM of fragmented mitochondria with irregular swelling and decreased cristae (arrowhead). (*n* = 8, *P < 0.05 vs. the LFD group, ^#^*P* < 0.05 vs. the HFD group).

Next, we investigated the effect of the Nrf2/ARE pathway on mtROS generation and mitochondrial injury in rats with HFD-induced obesity. Immunofluorescence analysis and Western blotting showed that the expression of Nrf2, HO-1, and NQQ-1 in HFD rats was upregulated compared with the expression of Nrf2, HO-1, and NQQ-1 in the LFD group, and the expression of Nrf2, HO-1, and NQQ-1 was further enhanced in HFD rats following treatment with tBHQ (Figures 9A–F). Further analysis demonstrated that mtROS generation (Figures 9G,I) and renal oxidative stress levels (Figures 9H,J) were enhanced in the kidneys of HFD rats compared with the kidneys of LFD rats; however, these enhancements were dramatically attenuated following tBHQ administration. Additionally, the degree of oxidative stress was also evaluated by detecting the MDA and GSH content and SOD activity in kidney tissue. Compared with the LFD group, the HFD group displayed a significant increase in MDA levels and a significant decrease in GSH levels and SOD activity (Figures 9K–M). tBHQ treatment significantly decreased MDA levels and increased the GSH levels and SOD activity.

Moreover, we also found that tBHQ treatment significantly alleviated the loss of ΔΨm in the kidneys of rats with HFD-induced obesity (Figures 9N,O). Furthermore, mitochondrial morphology was assessed by electron microscopy. In the LFD group, most of the tubular cells had elongated cylindrical-shaped mitochondria with organized cristae. In the HFD group, the tubular cells exhibited fragmented sphere-shaped mitochondria with irregular swelling and decreased cristae; however, the mitochondrial morphology was partially restored by tBHQ administration (Figure 9P).

## Discussion

Dyslipidemia is a common metabolic disorder and is known to be the most prevalent independent risk factor in patients with CKD. Since Moorhead first proposed the lipid nephrotoxicity hypothesis in 1982 (21), increasing evidence supports the hypothesis that ectopic lipid accumulation in the renal parenchyma can directly or indirectly lead to renal injury, which contributes to the development and progression of CKD (22). Consistently, we also verified increased lipid accumulation in renal proximal tubule cells *in vitro* and *in vivo*, and furthermore, we found that PA-induced cytotoxicity manifested as cell cycle arrest and decreased cell viability, cytoskeleton damage and cell apoptosis in HK-2 cells.

Although abnormal lipid accumulation can lead to lipotoxicity and cause cellular dysfunction, the precise mechanisms of renal injury are not fully understood. Importantly, proximal tubule cells seem to be more susceptible to lipid toxicity than other renal cells in the kidney because they require higher energy expenditure for massive tubular reabsorption, and thus, they critically depend on FAO. In the face of this demand, proximal tubule cells preferentially take up and utilize FA as a major energy source, likely because fatty acids produce 3 times more ATP than glucose (23). The β-oxidation of fatty acids occurs mainly in the mitochondria; hence, lipid-induced mitochondrial damage may be catastrophic for proximal tubule cells.

Mitochondria are essential organelles present in most cells that play a critical role in supplying the cell with metabolic energy and are known as the “cell powerhouse,” the core of cellular energy metabolism (24). In addition, mitochondria are highly dynamic organelles that continuously undergo fission and fusion processes to change their morphology, which is essential for mitochondrial quality control and maintaining normal cellular function (25, 26). In this study, we found that PA induced significant mitochondrial fragmentation; however, the mechanism of PA-induced mitochondrial fragmentation in HK-2 cells remains poorly understood. ROS production has been reported to be associated with mitochondrial dynamics, and increased ROS are implicated in inducing mitochondrial fragmentation, which in turn can promote ROS generation (27, 28). Moreover, previous studies have shown that mitochondrial fragmentation is closely related to apoptosis and suggest that mitochondrial fragmentation may be a driver of apoptosis (29–31). Our study found that preincubation with MitoTEMPOL to scavenge mitochondrial ROS significantly attenuated PA-induced mitochondrial fragmentation, suggesting that there is an interplay between ROS and mitochondrial fragmentation in HK-2 cells under high-lipid conditions.

Emerging evidence suggests that mitochondrial dysfunction plays a pivotal role in the development of CKD (32). Additionally, mitochondrial dysfunction is reportedly involved in obesity and obesity-related disorders, including obesity-linked kidney injury (33, 34), and preserving mitochondrial function has been shown to overcome lipotoxicity in the kidney (35). Importantly, mitochondria are the primary source of intracellular ROS production and are also potentially vulnerable to ROS (19). Mitochondrial dysfunction involves the overproduction of ROS, and excess ROS may induce oxidative damage to mitochondria, which forms a vicious cycle. Mitochondrial dysfunction, including excessive mitochondrial fission and mitochondrial membrane potential collapse, promotes the release of cytochrome c from the mitochondria to the cytoplasm, where cytochrome c activates caspase-9 and caspase-3 and finally triggers cellular mitochondrial-dependent apoptosis pathways (36). In the present study, we found that high lipid conditions induced mitochondrial fragmentation, loss of ΔΨm and increased mitochondrial ROS generation *in vitro* and *in vivo*. Furthermore, pretreatment with MitoTEMPOL significantly attenuated PA-induced mitochondrial ROS generation, mitochondrial injury, cytoskeletal damage, and cell apoptosis in HK-2 cells. These results demonstrated that mtROS are involved in mitochondrial dysfunction and cell injury in PA-induced HK-2 cells.

As the master regulator of cellular redox homeostasis, the Nrf2/ARE signaling pathway plays a crucial role in regulating the cellular oxidative stress response and metabolism (37). In kidney research, genetic activation of Nrf2 signaling has been reported to markedly suppress the onset of diabetes (38), and knocking out Nrf2 increases renal oxidative stress and accelerates renal injury in STZ-induced diabetic mice (17). However, the role of Nrf2 in tubular oxidative stress and mitochondrial function in hyperlipidemia-induced renal injury is not fully understood. In our study, we found that the Nrf2/ARE signaling pathway was activated in the kidneys of rats with HFD-induced obesity and in HK-2 cells exposed to high lipid levels; however, silencing Nrf2 accelerated PA-induced mitochondrial ROS generation, mitochondrial dysfunction, cytoskeletal damage and cell apoptosis in HK-2 cells. Importantly, activation of Nrf2 with tBHQ significantly attenuated kidney injury by ameliorating mitochondrial ROS generation and oxidative stress levels and improving mitochondrial dysfunction in the kidneys of rats with HFD-induced obesity. Our findings are in line with a previous study that showed that activation of Nrf2 with tBHQ significantly alleviated contrast-induced nephropathy in rats by activating the downstream antioxidative pathway (39). Additionally, our study showed that MitoTEMPOL effectively eliminated the negative effect of Nrf2 silencing on mitochondrial dysfunction and cell injury in PA-induced HK-2 cells, indicating that the protective role of the Nrf2/ARE signaling pathway on PA-induced HK-2 cells is associated with ameliorating mtROS-mediated mitochondrial dysfunction. Furthermore, tBHQ significantly decreased the levels of lipid accumulation in proximal tubule cells in the kidney tissues of HFD rats, the potential mechanism of which may be involved in improving mitochondrial function through ameliorating mtROS-mediated mitochondrial dysfunction, thereby enhancing mitochondrial fatty acid oxidation capacity and alleviating lipid nephrotoxicity. This process needs to be further elucidated in future studies. However, the limitation of this study is the lack of a separate tBHQ intervention group in animal experiments, further *in vivo* studies might be necessary. Collectively, these results suggest that the Nrf2/ARE-mediated antioxidant signaling pathway prevents renal tubular epithelial cell injury and apoptosis under high lipid exposure by reducing mitochondrial ROS and subsequently preserving mitochondrial function. Interestingly, there is some evidence for an association of Nrf2 with mitochondrial function, and recent studies have shown that Nrf2 affects mitochondrial function by regulating mitochondrial redox homeostasis, mitochondrial biogenesis or mitophagy activation (40). Li et al. showed that Nrf2 contributed to the restoration of mitophagy and mitochondrial quality by regulating PINK1/Parkin transcription, thereby attenuating hyperglycemia-induced tubular injury and apoptosis (41). However, the exact mechanism by which Nrf2 regulates tubular mitochondrial function in hyperlipidemia-induced renal injury remains to be delineated in further research.

## Conclusion

**FIGURE 10:**
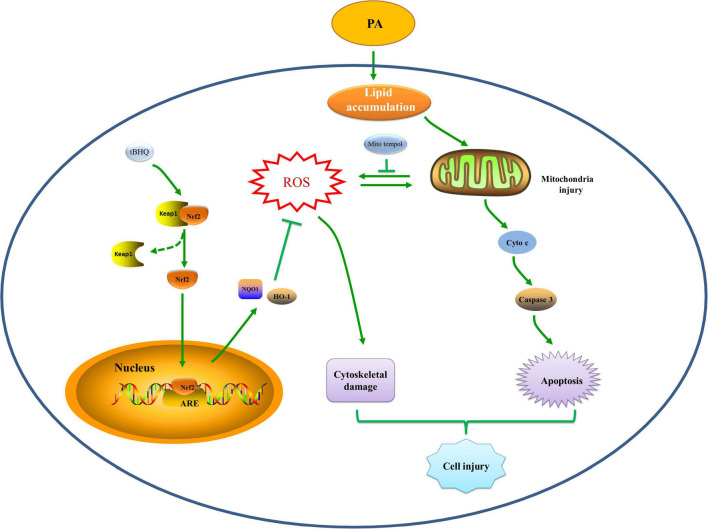
Hypothetical mechanisms by which the Nrf2/ARE signaling pathway prevents renal tubular cell injury by decreasing mitochondrial ROS production, preserving mitochondrial function, and ameliorating cytoskeletal damage and cell apoptosis under high lipid conditions. PA exposure induces an abnormal accumulation of lipids, which may induce mitochondrial injury accompanied by a reduction in ΔΨm, fragmented mitochondrial accumulation, and mitochondrial apoptotic pathway activation, which eventually leads to cell apoptosis. Mitochondria are the major source of intracellular ROS production and are also vulnerable to ROS, and mitochondrial damage can lead to excessive mtROS generation, which can induce cytoskeletal damage or further promote mitochondrial injury and apoptosis. The Nrf2/ARE signaling pathway is activated to reduce mtROS production, subsequently protecting against damaged mitochondria and attenuating cytoskeletal damage and cell apoptosis, thereby attenuating hyperlipidemia-induced tubular injury.

In summary, we provided evidence showing that hyperlipidemia induced Nrf2/ARE antioxidant signaling activation, mitochondrial dysfunction, mtROS generation, and cell injury. Additionally, the Nrf2/ARE-mediated antioxidant response prevented HK-2 cell apoptosis and tissue damage after high lipid media intervention by decreasing mitochondrial ROS production and subsequently preserving mitochondrial function, attenuating cytoskeletal damage and cell apoptosis. The hypothetical cellular and molecular events by which the Nrf2/ARE signaling pathway protects against palmitic acid-induced renal tubular epithelial cell injury are summarized in Figure 10. Thus, possible therapeutic strategies aimed at upregulating Nrf2/ARE antioxidant signaling may attenuate tubular epithelial cell injury against kidney injury in CKD with hyperlipidemia.

## Data availability statement

The original contributions presented in this study are included in the article/supplementary material, further inquiries can be directed to the corresponding author/s.

## Ethics statement

This animal study was reviewed and approved by the Ethics Committee of Chongqing Medical University.

## Author contributions

HG and X-GD conceived and designed the experiments. X-SJ and M-YC performed the experiments. X-SJ, QZ, X-GD, and M-YC analyzed the data. M-LL, Y-FX, QS, and X-JL contributed reagents, materials, and analysis tools. X-SJ, HG, and X-GD wrote the manuscript. All authors contributed to the article and approved the submitted version.
